# Binding of Cyclic Di-AMP to the Staphylococcus aureus Sensor Kinase KdpD Occurs via the Universal Stress Protein Domain and Downregulates the Expression of the Kdp Potassium Transporter

**DOI:** 10.1128/JB.00480-15

**Published:** 2015-12-14

**Authors:** Joana A. Moscoso, Hannah Schramke, Yong Zhang, Tommaso Tosi, Amina Dehbi, Kirsten Jung, Angelika Gründling

**Affiliations:** a Section of Microbiology and MRC Centre for Molecular Bacteriology and Infection (CMBI), Imperial College London, London, United Kingdom; b Center for Integrated Protein Science (CiPSM), Department of Biology I, Microbiology, Ludwig-Maximilians-Universität München, Martinsried, Germany

## Abstract

Nucleotide signaling molecules are important intracellular messengers that regulate a wide range of biological functions. The human pathogen Staphylococcus aureus produces the signaling nucleotide cyclic di-AMP (c-di-AMP). This molecule is common among Gram-positive bacteria and in many organisms is essential for survival under standard laboratory growth conditions. In this study, we investigated the interaction of c-di-AMP with the S. aureus KdpD protein. The sensor kinase KdpD forms a two-component signaling system with the response regulator KdpE and regulates the expression of the *kdpDE* genes and the *kdpFABC* operon coding for the Kdp potassium transporter components. Here we show that the S. aureus KdpD protein binds c-di-AMP specifically and with an affinity in the micromolar range through its universal stress protein (USP) domain. This domain is located within the N-terminal cytoplasmic region of KdpD, and amino acids of a conserved SXS-X_20_-FTAXY motif are important for this binding. We further show that KdpD2, a second KdpD protein found in some S. aureus strains, also binds c-di-AMP, and our bioinformatics analysis indicates that a subclass of KdpD proteins in c-di-AMP-producing bacteria has evolved to bind this signaling nucleotide. Finally, we show that c-di-AMP binding to KdpD inhibits the upregulation of the *kdpFABC* operon under salt stress, thus indicating that c-di-AMP is a negative regulator of potassium uptake in S. aureus.

**IMPORTANCE** Staphylococcus aureus is an important human pathogen and a major cause of food poisoning in Western countries. A common method for food preservation is the use of salt to drive dehydration. This study sheds light on the regulation of potassium uptake in Staphylococcus aureus, an important aspect of this bacterium's ability to tolerate high levels of salt. We show that the signaling nucleotide c-di-AMP binds to a regulatory component of the Kdp potassium uptake system and that this binding has an inhibitory effect on the expression of the *kdp* genes encoding a potassium transporter. c-di-AMP binds to the USP domain of KdpD, thus providing for the first time evidence for the ability of such a domain to bind a cyclic dinucleotide.

## INTRODUCTION

The Gram-positive bacterium Staphylococcus aureus is a commensal organism, with 20% of individuals being persistently and 30% being intermittently colonized (1). It is also a versatile pathogen, causing infections ranging from minor skin infections to severe invasive disease (1–3). A large arsenal of virulence factors, which includes secreted toxins, surface-attached adhesins, and other cell surface polymers such as teichoic acids and capsular polysaccharide, contributes to the pathogenesis of this organism (1, 4). The expression of these factors is intricately regulated during the infection process by a cell density-dependent quorum-sensing system as well as a range of transcription factors and two-component systems (5, 6).

S. aureus is also a major cause of food poisoning in Western countries (7). A common method used for food preservation is the reduction of water activity, which can be achieved under high-osmolarity conditions. However, early on, it was recognized that S. aureus can grow under conditions of low water activity, surviving in medium containing up to 20% (3.5 M) NaCl, while other bacteria, such as Pseudomonas aeruginosa or Escherichia coli, tolerate only up to 5% or 8.5% NaCl, respectively (8). This characteristic has been used to selectively propagate S. aureus on mannitol salt agar plates containing 7.5% NaCl, a concentration that is deleterious for many other bacteria (9).

When exposed to high-osmolarity conditions, bacteria rapidly accumulate potassium (K^+^) ions and so-called compatible solutes in order to survive (10). S. aureus has been shown to accumulate betaine and proline as compatible solutes (11, 12), and K^+^ uptake is mediated by two transport systems, namely, the Ktr and Kdp systems (13–15). The Ktr system is constitutively expressed in S. aureus (13), and based on its homology to the better-studied Ktr systems of Vibrio alginolyticus and Bacillus subtilis, it is thought to be a high- to moderate-affinity K^+^ uptake system (16–19). In Ktr systems, which are composed of a membrane component and a cytoplasmic gating component, K^+^ uptake is thought to occur with symport of Na^+^ ions (20). Two membrane components with seemingly redundant functions, KtrB and KtrD, are present in S. aureus, and they work together with one gating component referred to as KtrA or KtrC (14). It has been experimentally shown that the Ktr system is important for the growth of S. aureus under high-osmolarity and K^+^-limiting conditions (14, 21). Regarding the Kdp system, its function in S. aureus as a K^+^ uptake system was initially challenged (22, 23), but two recent studies have shown that S. aureus kdp mutants are unable to grow in defined medium under K^+^-limiting conditions (13, 14). This finding supports the notion that the Kdp system is also a bona fide K^+^ transport system in S. aureus.

The Kdp system has been studied best in E. coli, where it has been shown to be a high-affinity P-type ATPase K^+^ uptake system (24). From this work, it is also known that the Kdp K^+^ transporter is composed of four proteins: the ATPase KdpB and its chaperone KdpC; the actual K^+^ transport protein KdpA; and a small accessory membrane protein, KdpF, which is thought to aid in the stability or assembly of the complex (25). Unlike the Ktr system, K^+^ uptake is driven by ATP hydrolysis and a reversible phosphorylation step that leads to conformational changes within the Kdp transporter proteins (26). Furthermore, its expression is transcriptionally regulated by the two-component system KdpDE, where KdpD is the membrane sensor kinase and KdpE is the response regulator (27). In E. coli, KdpD activates and phosphorylates KdpE under K^+^-limiting or, to some extent, under high-osmolarity conditions (27, 28). Once phosphorylated, the cytoplasmic transcriptional factor KdpE binds the promoter region and activates transcription of the *kdpFABC* operon. Downstream of and overlapping with *kdpC* is the *kdpDE* operon. The *kdpFABC* transporter and *kdpDE* two-component system genes are also present in S. aureus. However, they are transcribed from two divergent promoters (23). Transcription of the *kdp* genes in S. aureus is strongly upregulated under high-osmolarity conditions caused by the addition of NaCl (13). Besides activating the expression of the *kdp* genes itself, the KdpDE two-component system has also been implicated in controlling the expression of the capsular genes and several other S. aureus virulence genes (13, 22, 23, 29). Noteworthy, while most S. aureus strains carry a single *kdp* operon, strains such as S. aureus MRSA252, Mu50, and N315 harboring staphylococcal cassette chromosome *mec* type II (SCC*mec* II) contain a second *kdp* operon (30).

Previous work from our laboratory identified components of both S. aureus K^+^ uptake systems as direct targets of the signaling nucleotide cyclic di-AMP (c-di-AMP) (21). More specifically, we have shown that c-di-AMP binds to KtrA (also referred to as KtrC), the cytoplasmic gating component of the Ktr system, and the sensor histidine kinase KdpD. c-di-AMP is a recently discovered signaling nucleotide commonly found in Gram-positive bacteria (31, 32). It is synthesized from two molecules of ATP by proteins with a diadenylate cyclase (DAC) domain and degraded to AMP and/or phosphoadenylyl adenosine (pApA) by phosphodiesterases with a DHH-DHHA1 domain or, as recently reported for Listeria monocytogenes, a protein with an HD domain (31, 33–36). Specifically, in S. aureus, c-di-AMP is synthesized by the diadenylate cyclase DacA and degraded by the DHH-DHHA1 domain-containing phosphodiesterase GdpP (37, 38). In previous work, we reported that depending on the growth phase, S. aureus has an intracellular c-di-AMP concentration of 2 to 8 μM (37, 38). A S. aureus gdpP mutant strain lacking the c-di-AMP phosphodiesterase has constitutively high levels of c-di-AMP of ∼50 μM (37, 38). The size of the *gpdP* mutant is 20% reduced compared to that of the wild-type (WT) strain, and the mutant has an increased resistance to beta-lactam antibiotics (37). The cyclase DacA and, hence, c-di-AMP production appear to be essential for the growth of S. aureus under standard laboratory conditions, as a *dacA* mutant strain could not be obtained (38).

In this study, we have further characterized the role of c-di-AMP in K^+^ homeostasis in S. aureus. More specifically, we investigated the binding and impact of c-di-AMP on the sensor kinase KdpD. Using truncated KdpD variants and variants with single-amino-acid substitutions, we show that c-di-AMP binds to a conserved amino acid motif within the universal stress protein (USP) domain of KdpD. We also show that the NaCl-dependent upregulation of the Kdp transporter genes is inhibited by high levels of c-di-AMP. Altogether, this suggest that c-di-AMP is a negative regulator of the Kdp system in S. aureus and likely also in other bacteria, as the conserved amino acid motif within the USP domain of KdpD is found in a range of Gram-positive bacteria.

## MATERIALS AND METHODS

### Bacterial strains and growth conditions.

Strains and plasmids used in this study are listed in Table 1. Unless otherwise stated, E. coli strains were grown aerobically in Luria-Bertani broth (LB), and S. aureus strains were grown in tryptic soya broth (TSB) at 37°C. When required, antibiotics were added as indicated in Table 1. For salt stress experiments, S. aureus strains were grown overnight in standard LB medium (containing 10 g/liter of tryptone, 5 g/liter of yeast extract, and 0.085 M NaCl). The next day, the cultures were back diluted to an optical density at 600 nm (OD_600_) of 0.05 in LB–0 M NaCl (10 g/liter of tryptone and 5 g/liter of yeast extract) or LB–1 M NaCl (10 g/liter of tryptone, 5 g/liter of yeast extract, and 1 M NaCl) and grown until the indicated time points.

**TABLE 1:** Bacterial strains used in this study

Strain	Relevant feature(s)^*a*^	Source or reference
Escherichia coli		
XL1-Blue	Cloning strain; Tet^r^; ANG127	Stratagene
BL21(DE3)	Strain for overproduction of proteins; ANG191	Novagen
T7IQ	Strain for overproduction of proteins; ANG2712	NEB
ANG1867	pET28b in E. coli; Kan^r^	Novagen
ANG2703	BL21(DE3)/pET28b-*kdpD*-FL(3–885)-His Kan^r^	21
ANG2694	XL1-Blue/pET28b-*kdpD*-NT(3–383)-His Kan^r^	This study
ANG2704	BL21(DE3)/pET28b-*kdpD*-NT(3–383)-His Kan^r^	This study
ANG2697	XL1-Blue/pET28b-*kdpD*-CT(492–885)-His Kan^r^	This study
ANG2707	BL21(DE3)/pET28b-*kdpD*-CT(492–885)-His Kan^r^	This study
ANG3119	XL1-Blue/pET28b-*kdpD*-KdpD(3–225)-His Kan^r^	This study
ANG3121	BL21(DE3)/pET28b-*kdpD*-KdpD(3–225)-His Kan^r^	This study
ANG3120	XL1-Blue/pET28b-*kdpD*-USP(225–383)-His Kan^r^	This study
ANG3122	BL21(DE3)/pET28b-*kdpD*-USP(225–383)-His Kan^r^	This study
ANG3166	XL1-Blue/pET28b-*kdpD2*-His Kan^r^	This study
ANG3167	BL21(DE3)/pET28b-*kdpD2*-His Kan^r^	This study
ANG3152	T7IQ/pVL847-Gn-GW-His-MBP-SACOL0066 Gen^r^ Cam^r^	This study
ANG3153	T7IQ/pVL847-Gn-GW-His-MBP-SACOL00556 Gen^r^ Cam^r^	This study
ANG3154	T71Q/pVL847-Gn-GW-His-MBP-SACOL1753 Gen^r^ Cam^r^	This study
ANG3155	T7IQ/pVL847-Gn-GW-His-MBP-SACOL1759 Gen^r^ Cam^r^	This study
ANG3117	XL1-Blue/pMALX(E) Amp^r^	39
ANG3175	XL1-Blue/pMALX(E)-MBP-*kdpD*(USP) Amp^r^	This study
ANG3177	BL21(DE3)/pMALX(E)-MBP-*kdpD*(USP) Amp^r^	This study
ANG3518	XL1-Blue/pMALX(E)-MBP-*kdpD*(USP)_S244A_ Amp^r^	This study
ANG3525	BL21(DE3)/pMALX(E)-MBP-*kdpD*(USP)_S244A_ Amp^r^	This study
ANG3520	XL1-Blue/pMALX(E)-MBP-*kdpD*(USP)_Y271A_ Amp^r^	This study
ANG3527	BL21(DE3)/pMALX(E)-MBP-*kdpD*(USP)_Y271A_ Amp^r^	This study
Staphylococcus aureus		
MRSA252	Wild-type MRSA strain; ANG3118	71
LAC*	Wild-type CA-MRSA strain (AH1263); ANG1575	72
ANG1961	LAC* *gdpP*::*kan* Kan^r^	37

a The following antibiotics were used: 30 μg/ml kanamycin (Kan), 100 μg/ml ampicillin (Amp), 20 μg/ml gentamicin (Gen), and 10 μg/ml chloramphenicol (Cam) for E. coli cultures and 90 μg/ml kanamycin for S. aureus cultures. MRSA, methicillin-resistant Staphylococcus aureus; CA-MRSA, community-associated methicillin-resistant Staphylococcus aureus.

### Strain and plasmid construction.

Primers used in this study are listed in Table 2. Plasmid pET28b-*kdpD-His* (strain ANG2703) contains the full-length *kdpD* gene from S. aureus strain LAC* with a C-terminal His tag, and its construction was described in a previous study (21). Plasmids pET28b-*kdpD*-NT, pET28b-*kdpD*-CT, pET28b-*kdpD*(KdpD), and pET28b-*kdpD*(USP) were constructed for the expression of truncated KdpD variants, producing the N-terminal cytoplasmic domain, the C-terminal cytoplasmic domain, and the KdpD or the USP domain. Plasmid pET28b-*kdpD-His* was used as the template, and primer pairs ANG1579/ANG1581, ANG1584/ANG1580, ANG1579/ANG1775, and ANG1776/ANG1581, respectively, were used to amplify the appropriate *kdpD* fragments. The resulting PCR products were digested with the restriction enzymes NcoI and EcoRI and inserted into plasmid pET28b. The plasmids were initially recovered in E. coli strain XL1-Blue and then isolated, sequenced, and introduced into E. coli BL21(DE3), yielding strains ANG2704, ANG2707, ANG3121, and ANG3122.

**TABLE 2:** Primers used in this study for PCR

Primer no.	Primer name	Sequence^*a*^
ANG1579	F-NcoI-FL-KdpD	GGGCCATGGCAAACACTGAATCGCTAAACATAGG
ANG1580	R-EcoRI-FL-KdpD	GGGGAATTCACGTCTCCTTCGTTAAAGTCTG
ANG1581	R-EcoRI-KdpD-NT	GGGGAATTCGCGAAACGTTTGCCTTTAGGACGATAG
ANG1584	F-NcoI-KdpD-CT	GGGCCATGGCCATTACTAAAAAGCAACTTTATCG
ANG1775	R-EcoRI-KdpD-KdpD	GGGGAATTCGGTTCTTTATCACTCATCAAGTCGGC
ANG1776	F-NcoI-KdpD-USP	GGGCCATGGAAAAAGTCCGACACAACCATAAAACGTC
ANG1777	1F-NcoI-FL-KdpD2	GGGCCATGGAAAGTACATATAAAAAAAGAGGGAAACTT
ANG1778	2R-NcoImut-KdpD2	GACATCGATACCGTGCGATAGAATTTCTTCTATATCC
ANG1779	3F-NcoImut-KdpD2	GAAATTCTATCGCACGGTATCGATGTCTGGACAAC
ANG1780	4R-EcoRI-FL-KdpD2	GGGGAATTCGGACGACCAATAATGTTGTTTTCATCCA
ANG1811	F-NheI-KdpD(USP)	GGGGCTAGCGAAAAAGTCCGACACAACCATAAAACGTC
ANG1812	R-HindIII-KdpD(USP)	GGGAAGCTTTCAGAAACGTTTGCCTTTAGGACGATAGGG
ANG2053	F-USPmutS244A	CACTCAAACCTCATATTGCTGTGGCAATTGCTGGGAGCATTTATAA
ANG2054	R-USPmutS244A	TTATAAATGCTCCCAGCAATTGCCACAGCAATATGAGGTTTGAGTG
ANG2055	F-USPmutY271A	CCTGTTTTTTTCGAATACATCTATAGCAATAGCAGTGAACTTCGCATGTTCTT
ANG2056	R-USPmutY271A	AAGAACATGCGAAGTTCACTGCTATTGCTATAGATGTATTCGAAAAAAACAGG

a Restriction sites in primer sequences are underlined.

For purification of the USP domain of S. aureus KdpD [KdpD^Sa^(USP)] as a maltose binding protein (MBP) fusion protein [MBP-KdpD(USP)], plasmid pMALX(E)-KdpD(USP) was constructed. To this end, the *kdpD* fragment coding for the USP domain region ranging from amino acids E225 to F383 was amplified by PCR using primer pair ANG1811/ANG1812. The resulting product was digested with the restriction enzymes NheI and HindIII and inserted into plasmid pMALX(E), which allows for an in-frame fusion to an N-terminal MBP tag (39). Plasmids pMALX(E)-KdpD(USP)_S244A_ and pMALX(E)-KdpD(USP)_Y271A_ were constructed for the expression and purification of MBP-KdpD(USP) variants with single-amino-acid substitutions in which S244 and Y271 were individually replaced by alanines. These plasmids were constructed by Quick-Change mutagenesis using primer pairs ANG2053/ANG2054 and ANG2055/ANG2056, respectively. Plasmids pMALX(E)-KdpD(USP), pMALX(E)-KdpD(USP)_S244A_, and pMALX(E)-KdpD(USP)_Y271A_ were initially recovered in E. coli strain XL1-Blue and then isolated, confirmed by sequencing, and subsequently introduced into E. coli strain BL21(DE3), yielding strains ANG3175, ANG3525, and ANG3527.

Plasmid pET28b-*kdpD2-His* was constructed for the production of KdpD2 from S. aureus strain MRSA252. To this end, the *kdpD2* gene was cloned into plasmid pET28b using the NcoI and EcoRI sites. As the *kdpD2* gene contained an internal NcoI site, the gene was initially amplified as two fragments by using primer pairs ANG1777/ANG1778 and ANG1779/ANG1780, and nucleotide T360 was mutated to a C in this step, which disrupts the internal NcoI site without changing the amino acid sequence. The two fragments were subsequently fused by overlap extension PCR using primer pair ANG1777/ANG1780 and cloned into plasmid pET28b by using the NcoI and EcoRI restriction enzymes. Plasmid pET28b-*kdpD2-His* was initially recovered in strain XL1-Blue and then isolated, confirmed by sequencing, and introduced into strain BL21(DE3) (yielding strain ANG3167) for protein production and the preparation of E. coli lysates.

The S. aureus COL genes with locus tags SACOL0066, SACOL0556, SACOL1753 (*usp1*), and SACOL1759 (*usp2*) were inserted by Gateway cloning into plasmid vector pVL847-Gn-GW. Plasmid pVL847-Gn-GW is derived from vector pVL847 (40), which was modified with a Gateway cloning cassette, and the *bla* gene (providing ampicillin resistance) was replaced with the *acc1* gene (providing gentamicin resistance). Vector pVL847-Gn-GW was obtained from Vincent Lee (University of Maryland) and allows for the production of N-terminally His-MBP-tagged fusion proteins. For the construction of plasmids pVL847-Gn-GW-SACOL0066, pVL847-Gn-GW-SACOL0556, pVL847-Gn-GW-SACOL1753 (*ups1*), and pVL847-Gn-GW-SACOL1759 (*usp2*), the pDONR221-derived plasmids containing the corresponding genes, obtained from the BEI Resource (NIAID, NIH), were used as donor plasmids in gateway reactions. Plasmids pVL847-Gn-GW-SACOL0066, pVL847-Gn-GW-SACOL0556, pVL847-Gn-GW-SACOL1753 (*ups1*), and pVL847-Gn-GW-SACOL1759 (*usp2*) were recovered in E. coli strain T7IQ, yielding strains ANG3152, ANG3153, ANG3154, and ANG3155, respectively. The sequences of all inserts were confirmed by sequencing and found to be error free.

### Preparation of E. coli whole-cell lysates.

For the preparation of E. coli whole-cell lysates, 5-ml cultures were grown overnight in LB at 30°C. To induce protein production, 1 mM IPTG (isopropyl-1-thio-β-d-galactopyranoside) was added directly to the cultures grown overnight, which were further incubated for 6 h at 30°C. Bacteria from the equivalent of a 1-ml culture with an OD_600_ of 3 were harvested by centrifugation and suspended in 100 μl of 40 mM Tris (pH 7.5)–10 mM MgCl_2_–100 mM NaCl buffer containing 2 mM phenylmethylsulfonyl fluoride (PMSF) (Sigma), 0.02 mg/ml DNase I (Sigma), and 0.5 mg/ml lysozyme (Sigma). Cells were lysed by 3 freeze-and-thaw cycles.

### Protein purification.

Proteins were purified from 1 or 2 liters of E. coli cultures grown at 37°C to an OD_600_ of 0.5 to 0.7, and protein production was induced with 0.5 mM or 1 mM IPTG overnight at 16°C. The next day, cells were collected by centrifugation, suspended in 20 ml of 50 mM Tris (pH 7.5)–150 mM NaCl–5% glycerol buffer, and lysed by using a French press. Lysates were cleared by centrifugation, and His-tagged proteins were purified by Ni-nitrilotriacetic acid (NTA) affinity and size exclusion chromatography, as previously described (21). The MBP fusion proteins expressed from plasmid pMALX(E) were purified over amylose resin (NEB BioLabs) and subsequently purified by size exclusion chromatography. To this end, the cleared bacterial lysates were incubated with 3 ml amylose resin, and the mixture was incubated for 60 min on a rotary wheel at 4°C. Next, the resin was allowed to settle in a column, the liquid was drained by gravity flow, and the resin was washed with 30 ml 50 mM Tris (pH 7.5)–150 mM NaCl–5% glycerol buffer. Proteins were eluted with 5 ml 50 mM Tris (pH 7.5)–150 mM NaCl–5% glycerol buffer containing 10 mM maltose. Further purification was achieved by size exclusion chromatography performed as described above, using a 50 mM Tris (pH 7.5)–200 mM NaCl–5% glycerol buffer system. Fractions containing the protein were pooled and concentrated using 10-kDa-cutoff Centricons (Millipore) and subsequently snap-frozen and stored at −80°C. The purity of the proteins was assessed by separating 10 μg of protein on 12% SDS gels and Coomassie staining. Protein concentrations were determined by using a bicinchoninic acid (BCA) kit (Pierce). For the Thermofluor experiment, cells expressing the wild-type or mutant MBP-KdpD(USP) fusion proteins were lysed in 50 mM Tris (pH 7.5)–500 mM NaCl–5% glycerol buffer containing 1 μg/ml DNase and a complete EDTA-free protease inhibitor cocktail (Roche). The proteins were purified over amylose resin by using 50 mM Tris (pH 7.5)–500 mM NaCl–5% glycerol buffer and further purified by size exclusion chromatography as described above.

### Protein stability analysis using a Thermofluor assay.

Purified proteins at a starting concentration of 100 μM were diluted to a final concentration of 10 μM in 20 μl of 40 mM Tris (pH 7.5)–10 mM MgCl_2_–100 mM NaCl containing 5× Sypro Orange dye (Life Technologies). The reactions were set up in triplicate in a 96-well plate, and thermal unfolding reactions were carried out with an Applied Biosystems OneStepPlus real-time PCR system. The temperature was increased by 1°C every 30 s from 25°C to 95°C, and fluorescence intensities were measured. To determine the background fluorescence, blank reactions were set up in the absence of protein. The data were analyzed using the Applied Biosystems StepOne Plus software. After subtraction of the blank values, the fluorescence readings were averaged and normalized to yield the unfolded-protein fraction as a function of temperature.

### DRaCALA.

A differential radial capillary action of ligand assay (DRaCALA) was performed as previously described (21, 41). Briefly, radiolabeled c-di-AMP was synthesized by incubating [α-^32^P]ATP (PerkinElmer) with the Bacillus thuringiensis DisA diadenylate cyclase enzyme (38). Next, 10-μl binding reaction mixtures were set up in 40 mM Tris (pH 7.5)–10 mM MgCl_2_–100 mM NaCl buffer containing ∼1 nM radiolabeled c-di-AMP and 9 μl of E. coli whole-cell lysates or purified proteins at a final concentration of 150 μM unless otherwise specified. The reaction mixtures were incubated at room temperature for 5 min. Two microliters of these reaction mixtures was then spotted onto a nitrocellulose membrane and air dried, and the radioactive signal was visualized and quantified using a Typhoon FLA 7000 phosphorimager. The fraction of bound nucleotide was determined as previously described (41). To determine the specificity of nucleotide binding, the reaction mixtures also contained 100 μM the specified, unlabeled nucleotides. For dissociation constant (*K_d_*) determinations, the MBP-KdpD(USP) protein was used at final concentrations ranging from 150 to 0.02 μM.

### Reverse transcription-qPCR.

S. aureus strain LAC* and the isogenic *gdpP* mutant strain were grown overnight in LB medium. The next day, the cultures were diluted to an OD_600_ of 0.05 in LB–0 M NaCl medium or LB–1 M NaCl medium and grown at 37°C to an OD_600_ of 0.7. Ten milliliters of the bacterial culture was then harvested, and the RNA was extracted as previously described (38). cDNA was synthesized by reverse transcription from 100 ng of RNA by using SuperScript III RNase H reverse transcriptase (Invitrogen) according to the manufacturer's specifications. Quantitative PCR (qPCR) was performed on an Applied Biosystems OneStepPlus real-time PCR system using TaqMan master mix and *gyrB* and *kdpA* 6-carboxyfluorescein (FAM)-labeled probes (Life Technologies). After confirming PCR efficiency for each gene, relative quantification (2^−ΔΔ*CT*^) was performed by calculating the cycle threshold (*C_T_*) variation between *kdpA* and *gyrB* and determining the variation of *kdpA* transcripts between salt stress (1 M NaCl) and no-salt-stress (0 M NaCl) conditions. Primer and probe sequences can be found in Table 3. Each experiment included three technical replicates, and statistically significant changes were determined by using the Student *t* test.

**TABLE 3:** Primers and probes used in this study for qPCR

Name	Purpose	Sequence
*gyrB*-F	Primer	CGCACGTACAGTGGTTGAAAA
*gyrB*-R	Primer	CGTGTTACTTCACGCGCTTTT
*gyrB*-P	Probe	ACGTGCCGCCATAATA
*kdpA*-F	Primer	AGCAGGTTTGTCAGCACTATTTACA
*kdpA*-R	Primer	AGGCGTTAAGCTATCATGCATGTT
*kdpA*-P	Probe	ATGCCGTCGTAATAAC

### Bioinformatics.

The modular architecture of KdpD was analyzed by using SMART (42), Pfam (43), and Phyre2 (44). KdpD homologues were identified by BLAST searchers (45), and phylogenetic trees were generated with Phylogeny.fr (46), using the maximum likelihood method. To determine which KdpD proteins have a USP domain similar to that of KdpD^Sa^(USP), the USP region from S. aureus was used as a query sequence in BLAST searches against all bacterial phyla with a KdpD homologue. Hits with a BLAST score of >60 and coverage of >80% were considered of interest, and the sequences and names of the top two species were retrieved to build a phylogenetic tree and further checked for the presence of a DacA homologue. When no DacA homologue was found, the presence or absence of DisA and GdpP was determined. To identify conserved amino acids potentially involved in c-di-AMP binding, the closest homologues of the S. aureus KdpD USP domain (amino acid residues E169 to R327) or the E. coli KdpD USP domain (residues R250 to D375) were used in two separate BLAST searches against the NCBI nonredundant protein sequence database. This yielded 470 hits with a minimum of 30% sequence identity, a maximum E value of 8 × 10^−4^, and 60% minimum sequence coverage for the S. aureus KdpD USP domain and 3,681 sequences with a minimum of 30% identity to the E. coli USP domain, of which the first 2,000 sequences were used for further analysis. For each subset of USP domain sequences, a multisequence alignment and a conserved sequence logo motif were generated with Clustal Omega (47). A structural model of the S. aureus KdpD(USP) domain was generated by using Phyre2 (44).

## RESULTS

### c-di-AMP binds to the USP domain of KdpD.

**FIG 1:**
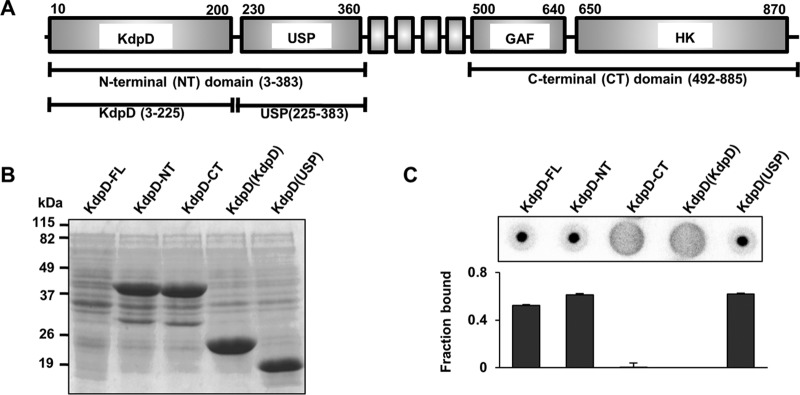
c-di-AMP binds the USP domain of KdpD^Sa^. (A) Schematic representation of the S. aureus KdpD protein and the truncated KdpD^Sa^ variants generated in this study. The sensor histidine kinase contains an N-terminal cytoplasmic region with KdpD and universal stress protein (USP) domains, a central four-transmembrane helix region, and a C-terminal cytoplasmic region with a GAF domain and a histidine kinase (HK) domain. The different regions are drawn to scale, with the amino acid numbers for the S. aureus KdpD protein indicated. (B) Coomassie-stained gel of E. coli lysates overproducing the different KdpD variants. Whole-cell lysates were prepared from E. coli strains expressing full-length KdpD (KdpD-FL) or the N-terminal cytoplasmic domain, the C-terminal cytoplasmic domain, the KdpD domain, or the USP domain of KdpD. Proteins were separated on a 12% SDS gel and visualized by Coomassie staining. Of note, no clear overproduction was observed for the full-length KdpD protein. The sizes of protein bands are indicated on the left and are given in kilodaltons. (C) c-di-AMP binds to the USP domain of KdpD. DRaCALAs were performed by using radiolabeled c-di-AMP and the E. coli extracts described above for panel B. At least three independent experiments were performed. Representative DRaCALA spots are shown, and the average fraction-bound values and standard deviations from triplicates were determined and plotted as previously described (21, 41).

**FIG 2:**
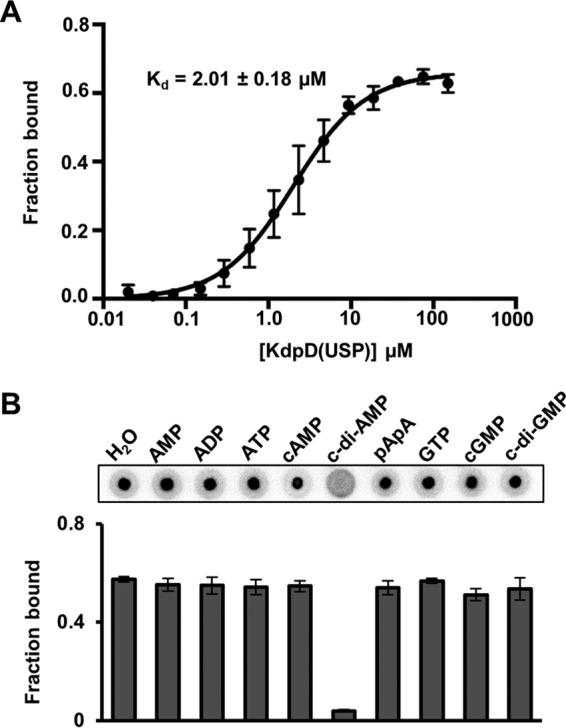
c-di-AMP binds specifically and with a *K_d_* of 2 ± 0.18 μM to the recombinant S. aureus MBP-KdpD(USP) protein. (A) c-di-AMP binding curve and *K_d_* determination using the purified MBP-KdpD(USP) fusion protein. DRaCALAs were performed with radiolabeled c-di-AMP and protein concentrations ranging from 0.02 to 150 μM. The average fraction-bound values and standard deviations from three independent experiments were plotted against the protein concentration, the binding curve was fitted by using one-site-specific binding nonlinear regression, and the *K_d_* value was determined as previously described (21, 41). (B) DRaCALAs were performed with the purified MBP-KdpD(USP) protein, radiolabeled c-di-AMP, and an excess (100 μM) of the indicated cold competitor nucleotide. Two independent experiments were performed. Representative DRaCALA spots are shown, and the average fraction-bound values and standard deviations from triplicates are plotted.

In previous work, the S. aureus sensor histidine kinase KdpD (here referred to as KdpD^Sa^) was identified as a c-di-AMP binding protein (21). Similar to the KdpD protein from E. coli (KdpD^Ec^), KdpD^Sa^ has a complex modular architecture with an N-terminal cytoplasmic region containing a KdpD domain and a USP domain, which is followed by four transmembrane helices and a C-terminal cytoplasmic region that harbors a putative GAF domain and a histidine kinase (HK) domain (Fig. 1A). To investigate which domain of KdpD^Sa^ interacts with c-di-AMP, full-length KdpD as well as truncated variants comprising only the N-terminal domain, the C-terminal domain, or the KdpD and USP domains were produced in E. coli. While no clear overexpression was observed for the full-length membrane-embedded KdpD^Sa^ protein, all other variants were overproduced in E. coli, and protein bands of the expected size were clearly visible in whole-cell lysates (Fig. 1B). The ability of the truncated variants to bind to c-di-AMP was assessed by a differential radial capillary action of ligand assay (DRaCALA) (21, 41) using whole-cell lysates and radiolabeled c-di-AMP. As expected, full-length KdpD^Sa^ interacted with c-di-AMP (Fig. 1C). No interaction between c-di-AMP and the C-terminal domain was observed, but the N-terminal part of the protein and, more specifically, the USP domain retained the ability to bind c-di-AMP (Fig. 1C). To confirm the interaction between c-di-AMP and the USP domain of KdpD^Sa^, the *kdpD*^Sa^ DNA fragment coding for the KdpD(USP) domain (amino acids E225 to F383) was cloned into vector pMALX(E), and the recombinant MBP-KdpD(USP) fusion protein was purified over amylose resin, followed by size exclusion chromatography. c-di-AMP interacted with the purified MBP-KdpD(USP) protein with a dissociation constant (*K_d_*) of 2 ± 0.18 μM (Fig. 2A), and binding was specific, as only an excess of unlabeled c-di-AMP but none of the other nucleotides tested was able to compete for binding with radiolabeled c-di-AMP (Fig. 2B). Taken together, these results show that c-di-AMP binds specifically to the USP domain of KdpD^Sa^.

### KdpD2 but not other USP domain proteins of S. aureus interacts with c-di-AMP.

**FIG 3:**
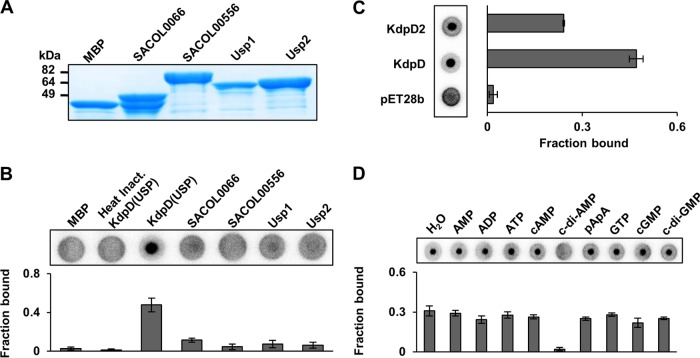
c-di-AMP binds to the USP domain of KdpD^Sa^ proteins but not to other USP domain-containing proteins. (A) S. aureus strain COL USP domain-containing proteins SACOL1753 and SACOL1759, together with SACOL0066 and SACOL0556, which have homology to USP domains, were purified as N-terminal His-MBP fusion proteins, and a Coomassie-stained gel loaded with 10 μg of the different purified proteins is shown. (B) DRaCALAs were performed with radiolabeled c-di-AMP and 150 μM concentrations of the different purified MBP-USP domain fusion proteins as well as the purified MBP-KdpD(USP) protein as a positive control or heat-inactivated MBP-KdpD(USP) and the MBP protein alone as negative controls. Fraction-bound values were determined, and average values and standard deviations from triplicates were plotted. (C) c-di-AMP interacts with KdpD2, a second KdpD protein found in a subset of S. aureus stains. Whole-cell lysates were prepared from E. coli BL21(DE3) strains containing the empty plasmid vector (pET28b) or expressing the full-length KdpD protein from S. aureus strain LAC* or the KdpD2 protein from S. aureus strain MRSA252. These lysates were used in DRaCALAs with radiolabeled c-di-AMP, representative spots are shown, and the average fraction-bound values with standard deviations from triplicates are plotted. (D) c-di-AMP interacts specifically with KdpD2. DRaCALAs were performed with an E. coli lysate expressing the full-length KdpD2 protein, radiolabeled c-di-AMP, and 100 μM the indicated cold competitor nucleotide. Representative spots are shown, and fraction-bound values were determined. Average values and standard deviations of data from three technical replicates are plotted.

After establishing that c-di-AMP binds to the USP domain of KdpD^Sa^, we wanted to test if c-di-AMP could also bind to other S. aureus proteins carrying USP domains. Using the protein sequences of the annotated USPs from E. coli (UspA, UspC, UspD, UspE, UspF, and UspG) and B. subtilis (NhaX and YxiE), BLAST searches were carried out against the S. aureus COL genome sequence. Two proteins, SACOL1753 and SACOL1759, consisting of a single USP domain, were identified and are referred to, as described in a previous study (48), as the Usp1 and Usp2 proteins, respectively. In addition to this, portions of SACOL0066 and SACOL0556 also aligned with 36% and 38% identity to the B. subtilis YxiE and E. coli UspD proteins, respectively. SACOL0066 is a 33-amino-acid-long oligopeptide, and tertiary-structure predictions revealed its closest match to be the UspE protein from Proteus mirabilis. SACOL0556 is a predicted heat shock chaperone belonging to the Hsp33 family of proteins that sense the redox state of the cell (49). A region of 55 amino acids in this protein aligned with the primary structure of UspD from E. coli, covering 35% of its sequence. Despite the lower levels of similarity of SACOL0066 and SACOL0566 to USPs, they were also included in the analysis. To investigate if SACOL0066, SACOL0566, Usp1 (SACOL1753), or Usp2 (SACOL1759) was able to bind c-di-AMP, we used an S. aureus ORFeome expression library available in the laboratory and purified the four USP domain-containing or related proteins as His-MBP fusions proteins (Fig. 3A). As assessed by DRaCALA and as shown in Fig. 3B, none of the purified His-MBP-USP fusion proteins was able to bind radiolabeled c-di-AMP under the conditions tested.

It was previously noted that S. aureus strains harboring SCC*mec* II contain a second *kdp* operon (30). For instance, S. aureus strain MRSA252 contains a second *kdpD* gene (locus tag SAR0069), and the encoded KdpD2 protein shares 58% identity with KdpD^Sa^ (locus tag SAR2166 in MRSA252). To test if KdpD2 can bind c-di-AMP, the gene was cloned into vector pET28b, and the production of full-length membrane-embedded KdpD2 was induced. Whole-cell lysates were prepared and used in DRaCALAs with radiolabeled c-di-AMP. This analysis revealed that the KdpD2 protein was also able to bind c-di-AMP (Fig. 3C) and that this binding was specific, as only an excess (100 μM) of cold c-di-AMP but not of any other nucleotide tested could compete for binding (Fig. 3D). In summary, these data suggest that binding of c-di-AMP is specific to the USP domain found in KdpD proteins and not a common feature of all USPs found in S. aureus.

### S. aureus KdpD(USP)-like domains are found in Firmicutes and Proteobacteria.

**FIG 4:**
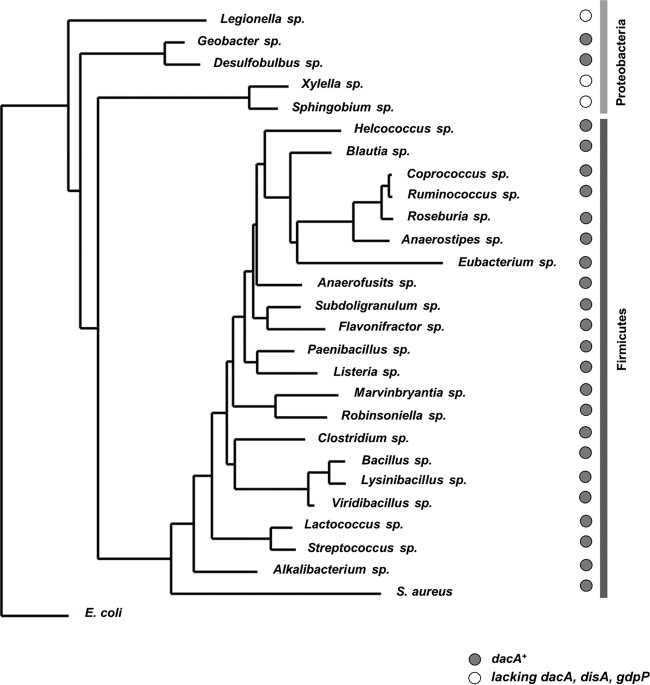
Phylogenetic tree of representative bacterial species with a KdpD homologue containing a USP domain similar to the one found in the S. aureus KdpD protein. KdpD homologues were retrieved by a BLAST search, and KdpD proteins with an S. aureus-like USP domain were identified and used to build a phylogenetic tree. Species that also harbor genes coding for a protein with a diadenylate cyclase (DAC) domain (i.e., proteins with homology to the S. aureus DacA or B. subtilis DisA protein) are denoted with a dark circle, while those lacking a diadenylate cyclase are indicated with a white circle.

The full-length KdpD^Ec^ and KdpD^Sa^ proteins share 26% identity, while their USP domains share only 20% identity. As E. coli does not produce c-di-AMP, one would not expect the KdpD^Ec^(USP) domain to interact with c-di-AMP. Therefore, we hypothesized that a subset of KdpD homologues that harbor a KdpD^Sa^-like USP domain would be present in strains that produce c-di-AMP. A BLAST search using the KdpD^Ec^ protein as a query sequence revealed that, as previously noted (50), KdpD is widespread in bacteria, and homologues were found in 10 out of the 22 phyla assayed, namely, in Acidobacteria, Actinobacteria, Bacteroidetes, Chlamydiae, Chloroflexi, Firmicutes, Planctomycetes, Proteobacteria, Spirochaetes, and Tenericutes. Interestingly, when the USP domain of KdpD^Sa^ was used as a query sequence, KdpD homologues were found only in Firmicutes and Proteobacteria (Fig. 4). Of these, all the species belonging to Firmicutes as well as Geobacter sp. and Desulfobulbus sp., which belong to the Deltaproteobacteria class, were found to also encode proteins with a DAC domain required for c-di-AMP production. Hence, this observation supports the notion that a subclass of KdpD proteins may have evolved to bind c-di-AMP.

### A conserved SXSX_20_-FTAXY motif in KdpD^Sa^(USP) is involved in c-di-AMP binding.

**FIG 5:**
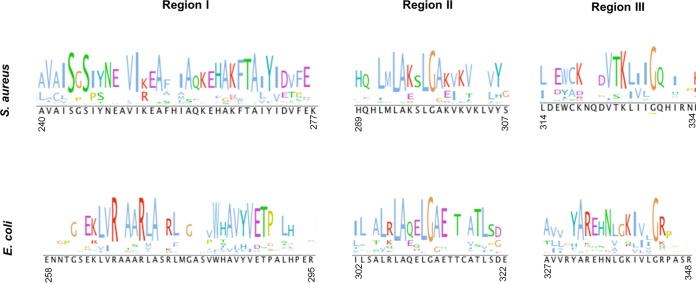
Identification of a conserved amino acid motif in the S. aureus KdpD(USP) domain. S. aureus and E. coli KdpD(USP) homologues were identified separately through BLAST searches. For each subset of USP domain sequences, a multisequence alignment and a conserved sequence logo motif were generated with Clustal Omega (47). Three regions (labeled I, II, and III) with highly conserved amino acid motifs were identified and are shown. While motifs II and III were similar in the S. aureus and E. coli KdpD(USP) domains, distinct conserved amino acids were observed in motif I. Specifically, a conserved SXS-X_20_-FTAXY motif spanning amino acids 244 to 271 was found in the USP domain of the S. aureus KdpD protein and its homologues.

**FIG 6:**
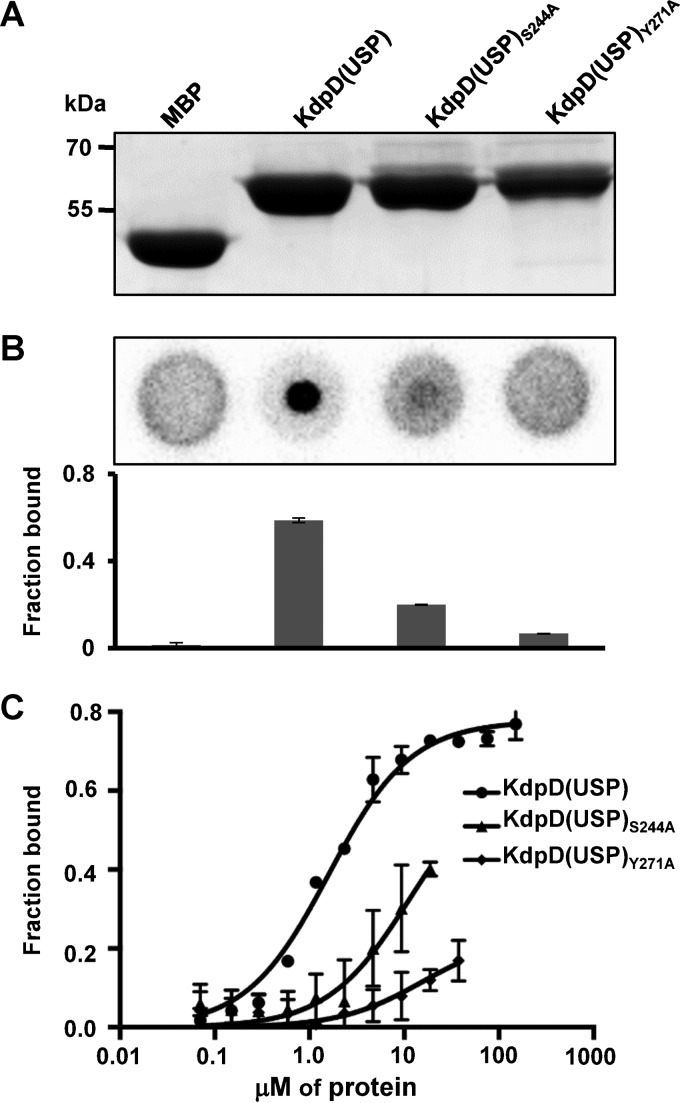
S. aureus KdpD(USP) variants with alanine substitutions in the conserved SXS-X_20_-FTAXY motif show reduced binding to c-di-AMP. (A) Coomassie-stained gel loaded with 10 μg of purified KdpD(USP) S244A and Y271A variants as well as MBP and MBP-KdpD(USP) as negative and positive controls, respectively. The KdpD(USP) variants were created by replacing the first conserved serine residue or final conserved tyrosine residues with alanines, and recombinant proteins were purified from E. coli as MBP fusion proteins. (B) DRaCALAs with radiolabeled c-di-AMP and 10 μM these four purified proteins. Two independent experiments were performed. Representative spots are shown, and the average fraction-bound values and standard deviations from three technical replicates are plotted. (C) c-di-AMP binding curve using the purified MBP-KdpD(USP) fusion protein and the S244A and Y271A variants. DRaCALAs were performed with radiolabeled c-di-AMP and protein concentrations ranging from 0.02 to 150 μM for wild-type MBP-KdpD(USP), 0.02 to 17.50 μM for the S244A variant, and 0.02 to 35 μM for the Y271A variant. The average fraction-bound values and standard deviations from three independent experiments were plotted against the protein concentration. *K_d_* values could not be determined for the two variants, as the saturation point could not be reached.

To elucidate a potential c-di-AMP binding site in the USP domain of KdpD^Sa^, we separately aligned the USP domain sequences of 470 KdpD^Sa^(USP) homologues and the top 2,000 KdpD^Ec^(USP) homologues and subsequently produced a logo motif. Three regions with conserved motifs were identified in both alignments (Fig. 5). The consensus sequences in regions II and III were similar in the E. coli and S. aureus KdpD(USP) domain alignments. However, the consensus in region I revealed the presence of a conserved SXS-X_20_-FTAXY motif in KdpD^Sa^(USP) homologues, whereas KdpD^Ec^(USP) displayed a conserved RXXXR-X_8_-WXAVY motif. We hypothesized that the SXS-X_20_-FTAXY motif, spanning amino acids 244 to 271 in KdpD^Sa^, might be required for the binding of c-di-AMP. To investigate this further, plasmids for the production of two MBP-KdpD^Sa^(USP) protein variants were generated, in which amino acid S244 or Y271 was replaced with alanines. The MBP-KdpD(USP)_S244A_ and MBP-KdpD(USP)_Y271A_ fusion proteins were produced in E. coli and purified along with the MBP and MBP-KdpD^Sa^(USP) control proteins (Fig. 6A). As assessed by DRaCALAs and *K_d_* determinations (Fig. 6B and C), the MBP-KdpD(USP)_S244A_ and MBP-KdpD(USP)_Y271A_ variants were impaired in c-di-AMP compared to the wild-type MBP-KdpD(USP) protein. To exclude the possibility that the c-di-AMP binding defect of the KdpD(USP) variants is due to an inherent instability or misfolding of the proteins, a Thermofluor experiment was performed, which is a fast and reliable technique to assess protein stability under a variety of conditions (51, 52). The experiment was performed in DRaCALA binding buffer using MBP as a control as well as freshly purified MBP-KdpD(USP) fusion proteins or the S244A and Y271A variants. All proteins display a single melting curve with low initial background fluorescence and a sharp thermal transition profile (see Fig. S1A in the supplemental material). This suggests that all proteins are properly folded. As the MBP-KdpD(USP) fusion proteins used for the Thermofluor experiments were purified under modified conditions (see Materials and Methods for details) and used prior to a frozen storage step, the c-di-AMP binding assays were repeated with the same proteins. Similar to the data shown in Fig. 6, c-di-AMP bound to the wild-type MBP-KdpD(USP) fusion protein, while a strong binding defect was observed for both single-amino-acid-substitution variants (see Fig. S1B in the supplemental material). Taken together, these data suggest that the S244 and Y271 amino acids in the conserved SXS-X_20_-FTAXY motif within the USP domain of KdpD^Sa^ are important for c-di-AMP binding.

### High levels of c-di-AMP inhibit upregulation of *kdpA* under salt stress.

**FIG 7:**
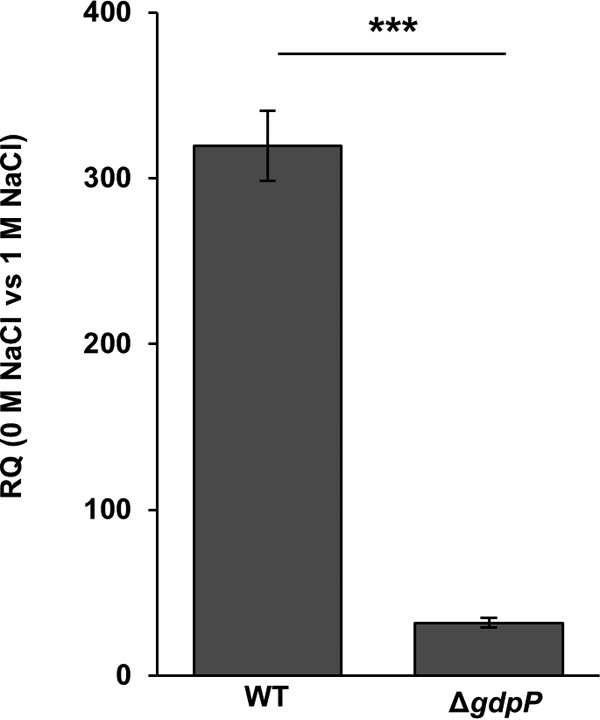
High levels of c-di-AMP inhibit expression of *kdpA*. Shown is the relative quantification (RQ) of transcript levels of *kdpA* measured by RT-qPCR for the wild-type S. aureus strain LAC* (WT) and an isogenic *gdpP* (Δ*gdpP*) mutant strain (with constitutively high levels of c-di-AMP), normalized to *gyrB* transcript levels. WT and mutant S. aureus strains were grown to an OD_600_ of 0.7 in LB medium without salt (0 M NaCl) or containing 1 M NaCl, and RNA was extracted and used for RT-qPCR experiments as described in Materials and Methods. Three independent experiments with triplicates were performed, and a representative result is shown. The data from one representative experiment are plotted and were analyzed by using a two-tailed Student *t* test. A statistically significant difference between the values was observed, with a *P* value of <0.01, and this is indicated by asterisks.

Previous work by Price-Whelan et al. showed that the KdpDE two-component system is required for the upregulation of the *kdpFABC* transporter genes under salt stress (13). To investigate the impact of c-di-AMP on the function of KdpD, reverse transcription-qPCR (RT-qPCR) experiments were carried out to monitor the transcript levels of the *kdpA* gene in wild-type (WT) S. aureus strain LAC* and an isogenic *gdpP* deletion strain, which has constitutively high levels of c-di-AMP (37, 38). These two strains were grown to mid-log phase in LB–0 M NaCl or LB–1 M NaCl medium to induce the expression of the *kdp* system. RNA was extracted, and transcript levels of the *kdpA* gene, normalized to the levels of the *gyrB* housekeeping gene, were compared. As expected, the levels of *kdpA* transcripts increased dramatically (320-fold) under salt stress in the WT strain (Fig. 7). In the *gdpP* mutant, the levels of *kdpA* transcripts under salt stress were only slightly increased (30-fold) compared to those in the WT strain (Fig. 7). This indicates that c-di-AMP binding to KdpD negatively affects the expression of the *kdpA* transporter genes. Combined with data from previous work, which suggested a function of c-di-AMP as a negative regulator of the S. aureus Ktr potassium transport system (21), this implicates this signaling nucleotide as a general negative regulator of potassium transport systems in S. aureus.

## DISCUSSION

To date, four c-di-AMP receptor proteins have been identified in S. aureus (21). Two of them, KtrA and KdpD, are involved in the regulation of the activity and expression, respectively, of the two main K^+^ uptake systems in S. aureus (13, 14). CpaA is a putative cation proton antiporter and therefore is likely also involved in ion transport across the membrane. The fourth protein, PstA, is a predicted signal transduction protein that has recently been shown to form homotrimers that coordinate c-di-AMP at the monomer-monomer interface (53, 54). PstA homologues have also been described as c-di-AMP target proteins in L. monocytogenes and B. subtilis (where it is named DarA), but their cellular function remains to be elucidated (55–57). In this study, we investigate the binding and impact of c-di-AMP on the function of one of these target proteins, namely, the sensor kinase KdpD. Our data suggest that c-di-AMP dampens the production of the Kdp K^+^ transport system under osmotic stress.

KdpD has an unusually complex molecular architecture compared to that of classical two-component sensor histidine kinases. It contains four cytoplasmic domains that could potentially bind c-di-AMP, namely, the N-terminal KdpD and USP domains and the C-terminal GAF and HK domains (Fig. 1A). Structure prediction of the KdpD domain revealed homology of this domain to NTPases (nucleoside triphosphatases), whereas GAF domains are commonly found in cyclic nucleotide cyclases and phosphodiesterases, thus suggesting binding to small nucleotide ligands. To be able to undergo autophosphorylation, HK domains are known to bind and hydrolyze ATP, and recent work by Lori et al. has shown that the sensor kinase CckA from Caulobacter crescentus binds c-di-GMP via a tyrosine residue in the HK domain to stimulate phosphatase activity and allow cell cycle progression (58). Finally, a subset of USP domain proteins is able to bind ATP (summarized in reference 59), and very recently, it was shown that the Mycobacterium tuberculosis USP Rv1636 can bind both cyclic AMP (cAMP) and ATP (60). Therefore, it was not possible to predict the c-di-AMP binding domain within the S. aureus KdpD protein. Using truncated and modified KdpD variants, we show that c-di-AMP interacts specifically with the USP domain of the S. aureus KdpD protein (Fig. 1, 2, and 6). This highlights that USP domains function in a larger number of nucleotide signaling networks than previously anticipated.

USP domains (Pfam accession number PF00582) are widespread among many organisms, including archaea, bacteria, fungi, plants, and even a few animals (43). They are generally associated with responses to different stresses, and bacterial species can have one or several USP domain-containing proteins. For example, E. coli contains, besides the USP domain in the sensor kinase KdpD, six USPs. UspA, UspC, UspD, UspF, and UspG are small proteins (∼130 amino acids) and have only a single USP domain, whereas UspE has two tandem USP domains (61, 62). The exact cellular function of USP domain proteins is unknown, but they are generally associated with survival under stress conditions. For instance, the E. coli UspA protein is regulated by the stringent response alarmone (p)ppGpp and produced under conditions of nutrient starvation, the addition of toxic agents, heat shock, and exposure to DNA damage reagents (63–67). Curiously, UspC has been shown to interact with the USP domain of E. coli KdpD under salt stress conditions (68).

Among the USPs that are able to bind ATP, a conserved G-X_2_-G-X_9_-G(S/T) amino acid motif is found (59). However, the actual functional consequence of nucleotide binding to these USP domains is not known. The mycobacterial USP domain protein Rv1636, which contains a typical ATP binding motif (59), was recently shown to bind ATP and cAMP, the latter with a 10-fold-higher affinity (60). The authors of that study suggested that this protein may acts as a “sink” for cAMP whereby the level of free cAMP in the cell is controlled by the amount of the Rv1636 protein. Those authors further hypothesized that Rv1636 may work as a module that couples cAMP signaling to the energy status of the cell.

**FIG 8:**
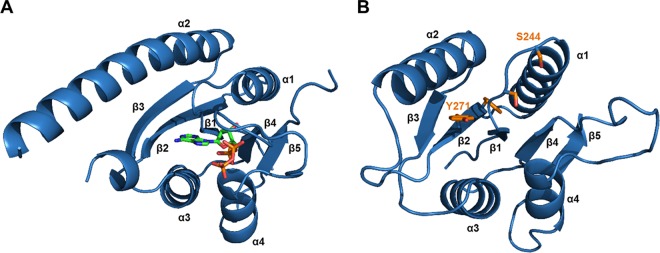
Structural model of the KdpD^Sa^(USP) domain. (A) Crystal structure of the MJ0577 USP from Methanocaldococcus jannaschii (PDB accession number 1MJH) bound to ATP. This protein has a canonical G-X_2_-G-X_9_-G(S/T) ATP binding motif, and residues important for binding are located between α1, β1, β2, and β2. (B) Structural model of the KdpD^Sa^(USP) domain generated with Phyre2 (44). The side chains of residues S244, S246, F267, and Y271 within the SXS-X_20_-FTAXY motif are shown in orange. Amino acids S244 and Y271 were shown to be important for c-di-AMP binding.

The USP domain of KdpD^Sa^ does not contain a recognizable ATP binding motif, but multiple-protein-sequence alignments revealed a region that is conserved in species that have a KdpD^Sa^-like USP domain and diverges from the same region of E. coli KdpD homologues (Fig. 5). Within this region, a conserved SXS-X_20_-FTAXY motif was identified and shown to be required for c-di-AMP binding (Fig. 6). While no structural information is available for the KdpD^Sa^(USP) domain, structures of >20 single-domain USPs have been determined (59). These proteins have a conserved fold with five β-strands that are sandwiched by four α-helices, as shown in Fig. 8A for the USP from Methanocaldococcus jannaschii. Typically, the ATP binding site is delineated by amino acids from α1, β1, β2, and β4. The KdpD^Sa^(USP) domain shares only 19% amino acid sequence identity with this protein; however, structural predictions suggest that it folds in a similar fashion (Fig. 8B). Interestingly, mapping of the SXS-X_20_-FTAXY motif onto the predicted structure suggests that the c-di-AMP binding pocket is delineated by residues in α1, β1, and β2, with the SXS residues being located in the loop between β1 and α1 and the FTAXY residues being located in β2. To some extent, this overlaps the ATP binding site. However, further conclusions can be drawn only when actual structural information on the KdpD^Sa^(USP) domain in complex with c-di-AMP is available. In this regard, recent work by Banerjee et al. is interesting to note, as those authors highlighted structural features in the mycobacterial USP Rv1636 which allows binding of cAMP and ATP but would prevent an interaction with c-di-AMP (60).

S. aureus strains with SCC*mec* II encode a second Kdp system, including a second KdpD protein, here referred to as KdpD2. It is currently not clear if this second K^+^ transporter and the corresponding two-component system are functional. We noticed, for instance, that the length of KdpD2 varied between strains due to single point mutations at the start of the *kdpD2* gene. However, we describe here that the KdpD2 protein from S. aureus strain MRSA252 is able to bind specifically to c-di-AMP (Fig. 3C and D). A protein sequence alignment revealed that the KdpD2 protein also contains the conserved SXS-X_20_-FTAXY motif within its USP domain. Furthermore, our data indicated that c-di-AMP binds solely to KdpD proteins and not to other USPs in S. aureus (Fig. 3A and B). As previously noted, S. aureus produces two readily recognizable single-domain USPs (locus tags SACOL1753 and SACOL1759 in the S. aureus COL genome) referred to as Usp1 and Usp2, respectively (48). Little is known about these USPs, but transcriptional upregulation and high Usp2 protein levels have been reported for two different mouse models of infection (48, 69). In addition to SACOL1753 (Usp1) and SACOL1759 (Usp2), we tested nucleotide binding to two other S. aureus proteins that have some homology to USPs, SACOL0066 and SACOL0556. None of these proteins was able to bind c-di-AMP in a physiologically relevant range, and consistent with this finding, none of these proteins contained the SXS-X_20_-FTAXY motif.

The data presented here indicate that high levels of c-di-AMP prevent the upregulation of the *kdp* transporter genes under salt stress (Fig. 7). Therefore, c-di-AMP binding to KdpD seems to impact, by an as-yet-unknown mechanism, KdpDE signaling, thus preventing the production of the Kdp K^+^ transporter. In light of this, it is conceivable that in the absence of stress, the production of the Kdp K^+^ transporter is inhibited until absolutely required by c-di-AMP binding to KdpD and that, under salt stress, this inhibition is relieved or bypassed. Similar to our observed inhibitory effect of c-di-AMP on the Kdp system, previous work by Bai et al. suggested that c-di-AMP impairs K^+^ uptake via the Ktr system in Streptococcus pneumoniae by binding to the cytoplasmic gating component named c-di-AMP binding protein (CabP) (70). It is of note that this c-di-AMP target does not contain a recognizable SXS-X_20_-FTAXY motif and hence must contain a different nucleotide binding motif. Taken together, this implicates c-di-AMP as a more general negative regulator of K^+^ uptake systems in Gram-positive bacteria.

In summary, here we identified the c-di-AMP binding site of KdpD^Sa^ to be located in the USP domain, and with this, we described for the first time the ability of such a domain to bind a cyclic dinucleotide. Additionally, we show that c-di-AMP prevents the expression of the *kdp* transporter genes under osmotic stress conditions. Since K^+^ is the main intracellular cation in living cells and is critical for enzyme function, pH homeostasis, and osmoregulation, this work further highlights the importance of c-di-AMP in regulating core biological functions in bacteria.

## Supplementary Material

Supplemental material
