# Supplementary material for: Binding of Cyclic Di-AMP to the Staphylococcus aureus Sensor Kinase KdpD Occurs via the Universal Stress Protein Domain and Downregulates the Expression of the Kdp Potassium Transporter
Source: J Bacteriol. 2015 Dec 14;198(1):98–110. doi: 10.1128/JB.00480-15 (PMC4686210; doi:10.1128/JB.00480-15)
Supplement: Supplemental material [file supp_198_1_98__index.html]

Binding of Cyclic Di-AMP to the Staphylococcus aureus Sensor Kinase KdpD Occurs via the Universal Stress Protein Domain and Downregulates the Expression of the Kdp Potassium Transporter — Supplemental material 

# Binding of Cyclic Di-AMP to the Staphylococcus aureus Sensor Kinase KdpD Occurs via the Universal Stress Protein Domain and Downregulates the Expression of the Kdp Potassium Transporter

## Supplemental material

- Supplemental file 1 -

  Fig. S1 (Thermal transition profiles and binding of MBP proteins)

  PDF, 248K
