# Supplementary material for: Binding of Cyclic Di-AMP to the Staphylococcus aureus Sensor Kinase KdpD Occurs via the Universal Stress Protein Domain and Downregulates the Expression of the Kdp Potassium Transporter
Source: J Bacteriol. 2015 Dec 14;198(1):98–110. doi: 10.1128/JB.00480-15 (PMC4686210; doi:10.1128/JB.00480-15)
Supplement: Supplemental material [file JB.00480-15_zjb999093760so1.pdf]

SUPPLEMENTAL MATERIAL

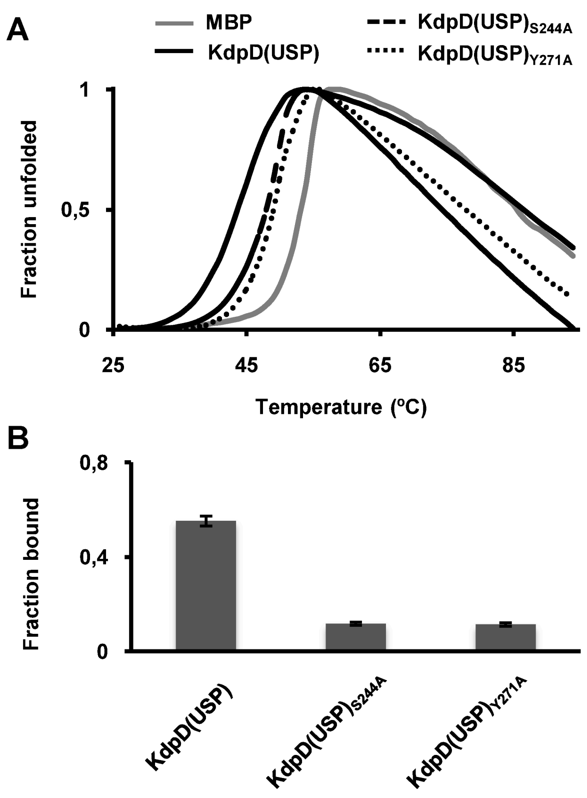


**Fig. S1. The S244A and Y271A MBP-KdpD(USP) variants, which show reduced c-di-AMP binding, are stable and folded.** (A) Thermal transition profiles of the MBP, MBP-KdpD(USP), MBP-KdpD(USP)$_{S244A}$ and MBP-KdpD(USP)$_{Y271A}$ proteins. A thermofluor experiment was performed in DRaCALA binding buffer with 10 µM of purified protein and 5x concentrated Sypro Orange dye using an Applied Biosystems OneStepPlus Real Time PCR System. The temperature was raised from 25°C to 95°C by 1°C every 30 seconds, the fluorescence intensity measured and the data analyzed using the Applied Biosystems StepOne Plus software. To determine the background fluorescence, blank reactions were set up in the absence of protein. After subtraction of the blank values, the fluorescence readings from three replicates were averaged and normalized to yield the unfolded protein fraction as a function of temperature. (B) DRaCALAs were performed with radiolabelled c-di-AMP and 25 µM of the purified proteins used in the thermofluor experiment. Average fraction bound values and standard deviation from two replicates with two spots each are plotted. The c-di-AMP binding ability is similar to that reported in Fig. 6B.
